# The TORC2 Component, Sin1, Controls Migration of Anterior Mesendoderm during Zebrafish Gastrulation

**DOI:** 10.1371/journal.pone.0118474

**Published:** 2015-02-24

**Authors:** Julien G. Dumortier, Nicolas B. David

**Affiliations:** 1 INSERM U1024, Paris, France; 2 CNRS UMR 8197, Paris, France; 3 IBENS, Institut de Biologie de l’Ecole Normale Supérieure, Paris, France; 4 Department of Physiology, Development and Neuroscience, University of Cambridge, Cambridge, United-Kingdom; Institute of Cellular and Organismic Biology, TAIWAN

## Abstract

TORC2 is a serine-threonine kinase complex conserved through evolution that recently emerged as a new regulator of actin dynamics and cell migration. However, knockout in mice of its core components Sin1 and Rictor is embryonic lethal, which has limited in vivo analyses. Here, we analysed TORC2 function during early zebrafish development, using a morpholino-mediated loss of function of *sin1*. Sin1 appears required during gastrulation for migration of the prechordal plate, the anterior most mesoderm. In absence of Sin1, cells migrate both slower and less persistently, which can be correlated to a reduction in actin-rich protrusions and a randomisation of the remaining protrusions. These results demonstrate that, as established in vitro, the TORC2 component Sin1 controls actin dynamics and cell migration in vivo. We furthermore establish that Sin1 is required for protrusion formation downstream of PI3K, and is acting upstream of the GTPase Rac1, since expression of an activated form of Rac1 is sufficient to rescue *sin1* loss of function.

## Introduction

Tor (Target Of Rapamycin) is a serine/threonine protein kinase, that is structurally and functionally conserved from yeast to mammals. It is present in two distinct protein complexes, named Tor Complex 1 (TORC1) and TORC2 [1]. In addition to Tor, these complexes share two common components, Lst8 (GβL) [2] and Deptor [3], while other components are distinct. While TORC1 includes Raptor [4] and PRAS40 [5], TORC2 contains Rictor (Rapamycin-insensitive companion of Tor, complex 2) [6,7], Sin1 (also known as Mapkap1) [8,9], PRR5 and PRR5L (proline rich 5 and proline rich 5 like, also known as Protor-1 and 2) [10,11]. At least Lst8 and Raptor for TORC1, and Rictor and Sin1 for TORC2, are critical for the complex assembly and/or for the binding of the Tor kinase to its substrates [7,12].

Much is known about the regulation and functions of TORC1, mainly due to its sensitivity to the natural compound, rapamycin. TORC1 is involved in many diverse cellular processes, including ribosome biogenesis, transcription and autophagy, but its canonical function is considered to be regulation of translation [13]. TORC2 on the other hand has been less studied but appeared in the past decade as a new regulator of the actin cytoskeleton and of cell migration. In yeast, TORC2 is required for the cell cycle dependent polarization of the actin cytoskeleton, through activation of PKC1, YPK2 (yeast protein kinase 2), and SLM (synthetic lethal with Mss4) [1,14,15]. In *Dictyostelium discoideum*, TORC2 loss of function leads to severe cell polarity defects and reduced chemotactic speed and directionality [16,17]. In this system, TORC2 acts in parallel to the PI3K pathway to activate PKB (Akt) which in turn regulates actin dynamics [18,19]. In mammalian cells, TORC2 plays a key role in neutrophil chemotaxis by regulating F-actin polarization and myosin II (MyoII) phosphorylation [20]. Contrary to the case in *Dictyostelium*, in mammalian cells this seems to be independent of Akt (PKB) but is mediated through PKC, which in turn modulates adenylyl cyclase and cAMP production. TORC2 was also shown to control Prostaglandin E2 dependent chemotaxis of mast cells [21], and more generally regulates actin dynamics in a number of cell lines [7,22]. In addition to PKC regulation, TORC2 modulates Rac1 activity, at least in part through activation of the Rac1 GEF P-Rex1 [23].

The role of TORC2 in controlling actin dynamics and cell migration is thus now well established in different cell types, and the molecular pathways involved are beginning to be unravelled. However, the functional importance of TORC2 dependent-migration remains unaddressed in metazoans. This most likely stems from the fact that, in mice, knock-out of the TORC2 components *sin1* or *rictor* leads to early embryonic lethality [6,8], which has precluded detailed analysis of these mutants. Tissue-specific knock-outs have revealed functions of TORC2 in different organs [24] However, since no major cell migrations take place in these adult tissues, it can be argued that the role of TORC2 in cell migration in vivo has still to be assessed.

Here, we used the zebrafish embryo to assess the role of TORC2 in controlling cell migrations in vivo. We show that loss of *sin1* function leads to defects in prechordal plate migration during gastrulation. Prechordal plate is composed of a group of cells that, during gastrulation, leads the forming embryonic axis. The prechordal plate migrates from the organiser (the node or shield in fish) to the animal pole, and later gives rise to the hatching gland [25–28]. Our analysis reveals that Sin1 controls both cell speed and persistence, and is essential for emission of actin-rich cell protrusions. This effect appears to be downstream of PI3K, and is mediated through Rac1.

## Results

### *sin1* is ubiquitously expressed during the first 24 hours of development

The TORC2 complex contains only two constituents which are both specific to the complex and essential to its function: Sin1 and Rictor. Three orthologues of *rictor* are present in the zebrafish genome complicating loss of function approaches, whereas *sin1* has only one. We thus focused our analysis on *sin1*. Zebrafish *sin1* ORF was amplified by RT-PCR (see Materials and Methods) and its expression profile was analysed by in situ hybridisation at different time during the first 24 hours of development. *sin1* mRNA appears maternally inherited in the egg (S1 Fig. A). From mid-blastula, when zygotic expression of the genome starts, to at least 24 hpf (hours post fertilisation), *sin1* is ubiquitously expressed (S1 Fig. B-E). During gastrulation however, it appears to be expressed at higher levels in the axial mesendoderm (S1 Fig. C and D).

### Partial loss of *sin1* function leads to embryonic axis widening

**Fig 1:**
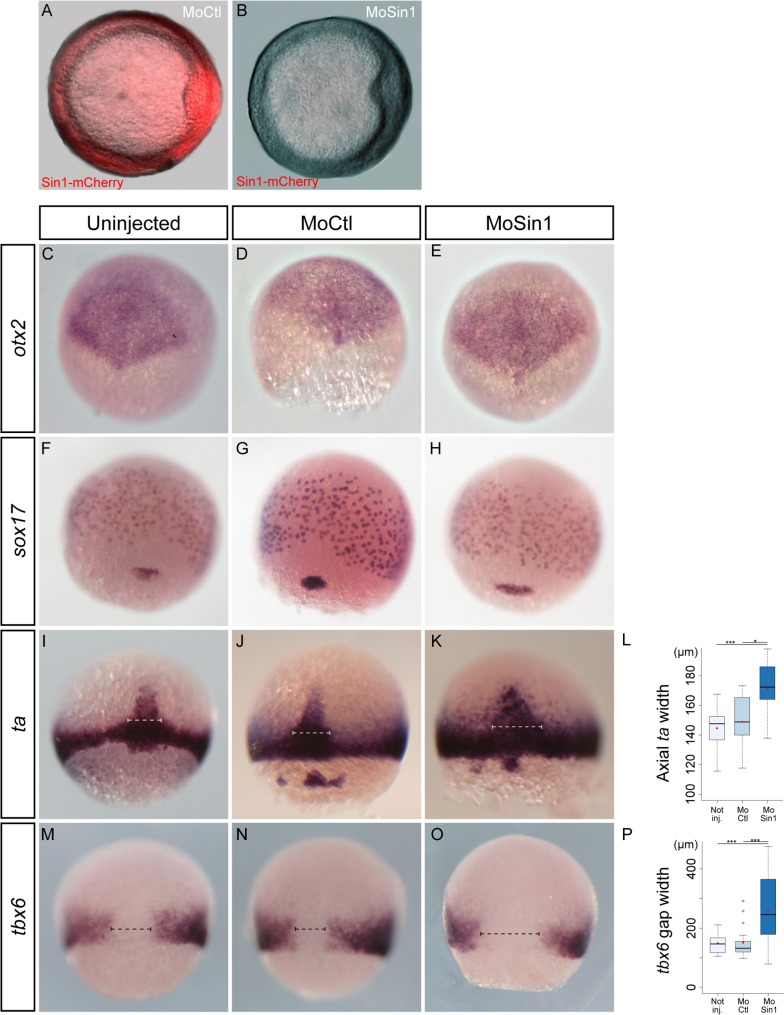
Loss of *sin1* function leads to embryonic axis enlargement. A-B. At the dose used in this analysis, *sin1* morpholino efficiently blocks translation of *sin1* RNAs. A control morpholino (A) or a morpholino targeting the translation initiation site of *sin1* (B) was co-injected at the 1-cell stage with mRNAs encoding a Sin1-mCherry fusion. Both images were acquired with the same exposure time. C-P. In situ hybridisations of genes expressed in the neuroectoderm (*otx2*), the endoderm (*sox17*), the mesoderm (*ta*) or the ventro-lateral mesoderm (*tbx6)*. Embryos are either un-injected, injected with a control morpholino or with *sin1* morpholino. They were fixed at mid-gastrulation. The three germ layers appear correctly formed. Mesoderm stainings nevertheless reveal a lateral widening of axial structures, quantified in L and P.

To assess the in vivo roles of TORC2, we analysed the effect of *sin1* loss of function during zebrafish development. Two independent morpholinos targeting both maternal and zygotic RNAs were used (one targeting the ATG start codon, the other targeting 5’UTR sequences; see Materials and Methods). Both morpholinos gave similar results and so will be collectively referred to as MoSin1, from now onwards. At high concentration MoSin1 blocks epiboly and leads to embryo lysis after 4 hours. This is consistent with the observed lethality of *sin1* and *rictor* knockout mice, which could be due to a role of TORC2 in cell growth and/or apoptosis [7,10]. To analyse the role of TORC2 during development, we lowered the quantity of morpholino injected (see Materials and Methods), most likely creating a hypomorphic situation, in which embryos can develop further. Using a Sin1-mCherry fusion construct, we checked that this dose of the MoSin1 still efficiently blocks translation of Sin1, while a control morpholino has no observable effect (Fig. 1A-B). At this dose, embryos show a delay in epiboly, but reach the end of gastrulation (tail bud stage) without any gross morphological defect. At that stage, they stop further development, and die several hours later, when control embryos are at mid-somitogenesis.

Specific markers for each of the three germ layers were used to further characterise the phenotype of morphant embryos during gastrulation. We analysed expression of *otx2* as an anterior neural marker [29], of *ta* (Brachyury homolog a, also known as *ntl*) as a mesodermal marker [30] and *sox17* as an endodermal marker [31]. These confirmed that, at mid-gastrulation, the overall organisation of the embryo is not affected by *sin1* loss of function (Fig. 1C-K). The *ta* staining nevertheless revealed a lateral widening of the notochord (dotted lines Fig. 1I-L; 148.88μm in non-injected embryos, n = 19; 144.50μm in MoCtl, n = 14; 171.40μm in MoSin1, n = 14; p_NI-MoCtl_ = 0.51, p_NI-MoSin1_ = 6.10^–5^, p_MoCtl-MoSin1_ = 6.10^–3^). Analysis of *tbx6* expression, which labels ventral and lateral mesoderm, confirmed this widening of the axial mesoderm, the dorsal gap of *tbx6* expression being 1.7 times wider in *sin1* morphants than in control embryos (Fig. 1M-P; 148.47μm in non-injected embryos, n = 14; 150.36μm in MoCtl, n = 19; 257.83μm in MoSin1, n = 22; p_NI/MoCtl_ = 0.90, p_NI/MoSin1_ = 3.10^–4^, p_MoCtl/MoSin1_ = 5.10^–4^).

### Prechordal plate migration is affected in absence of Sin1

**Fig 2:**
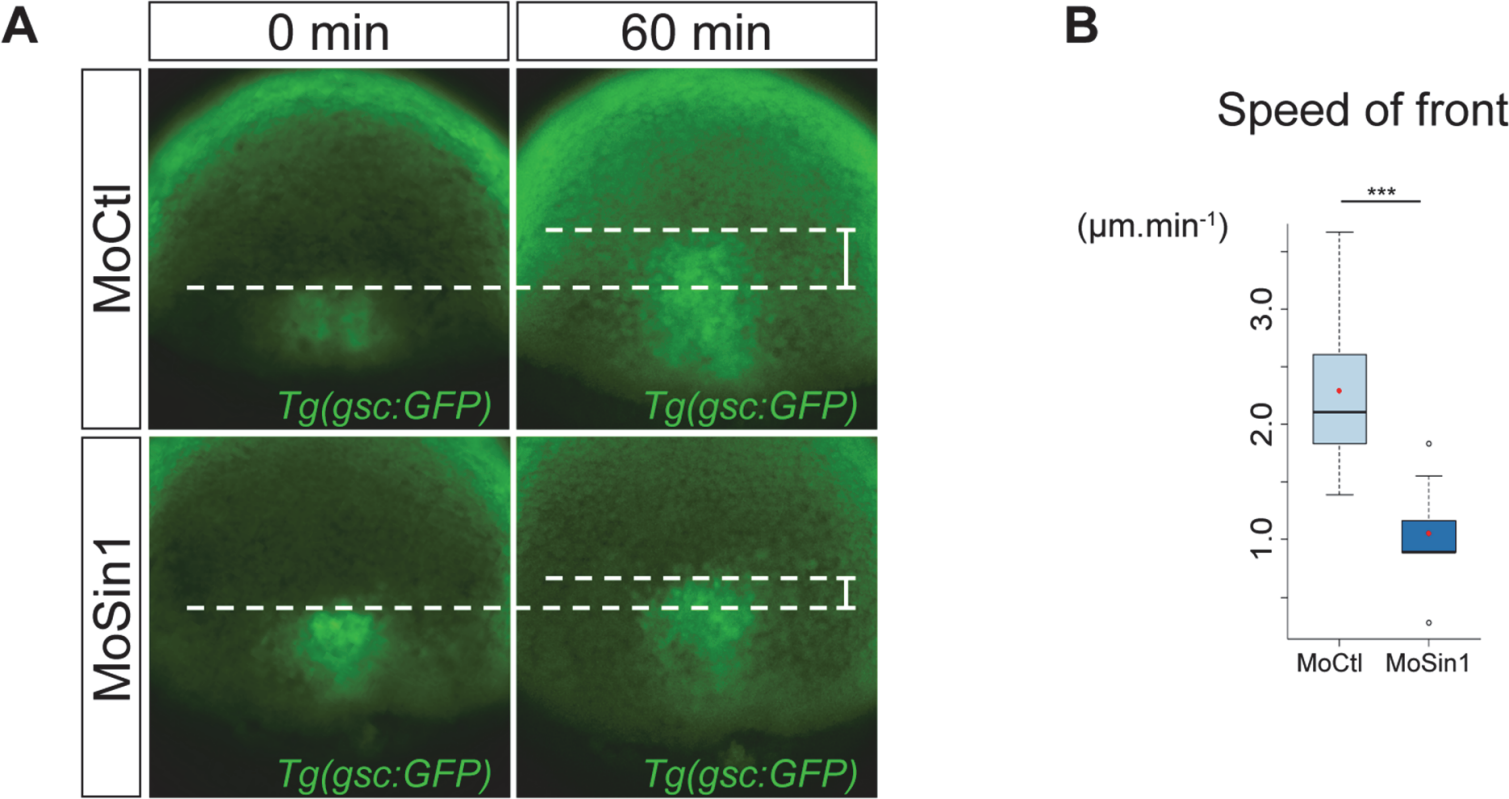
The prechordal plate migrates slower in *sin1* loss of function. (A) Progression of the front of the prechordal plate was monitored in *Tg(-1*.*8gsc*:*GFP)* embryos (see S1 Movie). In embryos injected with the *sin1* morpholino, the prechordal plate migrates twice slower than in embryos injected with a control morpholino. (B) Average speed of the front of the prechordal plate (distance travelled over one hour divided by 60 minutes), in embryos injected with a control morpholino or the *sin1* morpholino.

Widening of axial mesoderm is a classical hallmark of defective axis extension [32,33]. We therefore monitored axis elongation in *sin1* morphants during gastrulation. To do so, we took advantage of a transgenic line, *Tg(-1*.*8gsc*:*GFP)*, in which GFP is expressed under the control of *goosecoid* (*gsc*) regulatory elements [34]. In this line, prechordal plate, the anterior most axial mesoderm structure, and the notochord are GFP labelled. To analyse axis extension, we monitored progression of the front of the prechordal plate (Fig. 2A and S1 Movie). Because embryonic development arrests at the end of gastrulation in *sin1* morphants, we limited our analysis of plate migration to the first half of gastrulation, when the embryo develops properly (Fig. 1C-O). Over one hour, the front of the prechordal plate in *sin1* morphants covers half the distance it covers in embryos injected with a control morpholino (Fig. 2B, average speed in MoCtl: 2.29μm.min^-1^, n = 9 embryos; average speed MoSin1: 1.05μm.min^-1^, n = 11 embryos; p = 5.10^–4^).

**Fig 3:**
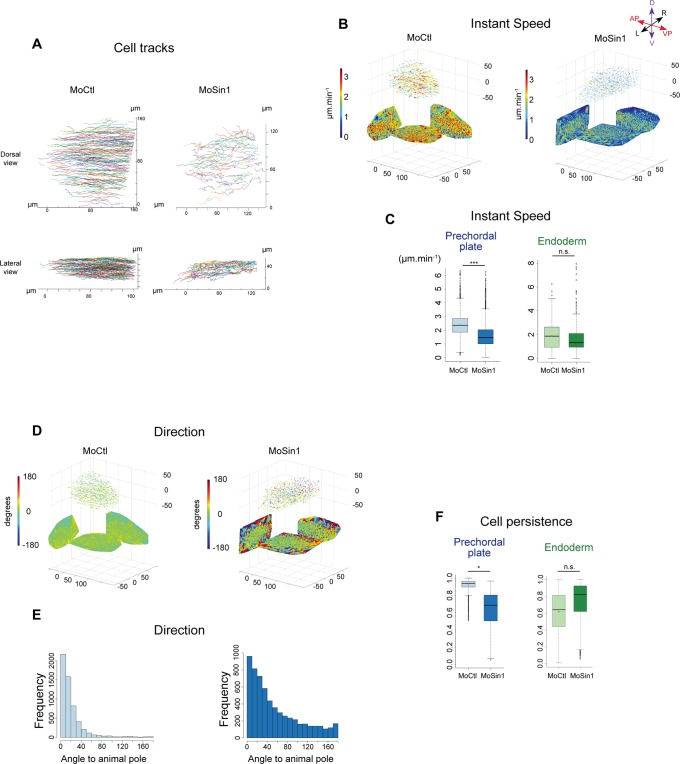
In absence of *sin1*, prechordal plate cells migrate slower and less persistently. (A) Tracks of prechordal plate nuclei in one representative embryo injected with a control morpholino or the *sin1* morpholino, dorsal and lateral views (see S2 Movie and S3 Movie). (B) Field of instantaneous speed in the prechordal plate (see Materials and Methods). Arrows indicate speed vectors, speed norm is colour-coded. Two-dimensional projections on the three planes have been plotted. (C) Instantaneous speed (measured on 2-minute intervals) of prechordal plate cells and endodermal cells, in embryos injected with the *sin1* morpholino or a control morpholino. Speed of prechordal plate cells is reduced in *sin1* loss of function, whereas speed of endodermal cells, used as a control, is not affected. (D) Same as in B, but colour-coding represents the direction of the movement. (E) Orientation of cell movements relative to the direction of the animal pole. In absence of *sin1*, cell movements are less directed towards the animal pole. (F) Cell persistence, measured as the ratio between the net displacement of the cell and the total length path over ten minute intervals. In absence of *sin1*, persistence of prechordal plate cells is reduced whereas persistence of endodermal cells is not.

The observed reduction in anterior-ward velocity of prechordal plate could be explained by at least two non-exclusive hypotheses: the first is that each cell migrates more slowy; the second is that cell movement is less oriented towards the animal pole, leading to a less persistent migration. To identify the cellular basis of prechordal plate retardation, we quantified cell movements within the plate. Histone-2B-mCherry labelled *Tg(-1*.*8gsc*:*GFP)* embryos were imaged every two minutes, during the first half of gastrulation. At each time point, a z-stack encompassing the whole prechordal plate was acquired. The movements of all prechordal plate cells were obtained by 3D tracking of their nuclei (Fig. 3A and S2 and S3 Movies) [35]. The instantaneous speed of prechordal plate cells (distance between two consecutive time points divided by time interval) appears strongly reduced in *sin1* loss of function (Fig. 3B-C, MoCtl: 2.38 μm.min^-1^, n = 5588 cell movements over 3 embryos; MoSin1: 1.59 μm.min^-1^, n = 6029 cell movements over 3 embryos, p = 7.10^–3^). To ensure that reduction in cell speed was specific to prechordal plate, we took advantage of the fact that *Tg(-1*.*8gsc*:*GFP)* embryos also express GFP in dorsal endodermal cells, which wander on the surface of the yolk syncytial layer during gastrulation [36]. Loss of *sin1* function does not affect the speed of endodermal cells (Fig. 3C and S4 Movie, MoCtl: 1.88μm.min^-1^, n = 443 cell movements over 3 embryos; MoSin1: 1.78μm.min^-1^, n = 794 cell movements over 3 embryos, p = 0.265, estimated power = 1).

In addition to cell speed, we analysed the orientation of cell movements. In absence of *sin1*, cell movements are less directed towards the animal pole (Fig. 3D-E, standard deviation to animal pole direction: 31.23º in MoCtl, 73.06º in MoSin1; p<2.2.10^–16^). Cells frequently changing direction, this loss of directionality leads to a strong reduction in cell persistence, persistence being the ratio between the net displacement of the cell and the total length path (Fig. 3F, Control: 0.92, n = 2782 over 3 embryos; Morphant: 0.66, n = 2461 over 3 embryos; p = 2.10^–2^). Again, we analysed endodermal cells as controls and found no significant reduction in their persistence (Fig. 3F, 3 control and 3 morphant embryos, p = 0.206, estimated power = 1). Sin1 thus controls both cell speed and cell directionality during prechordal plate migration.

### Sin1 controls cell protrusion formation

**Fig 4:**
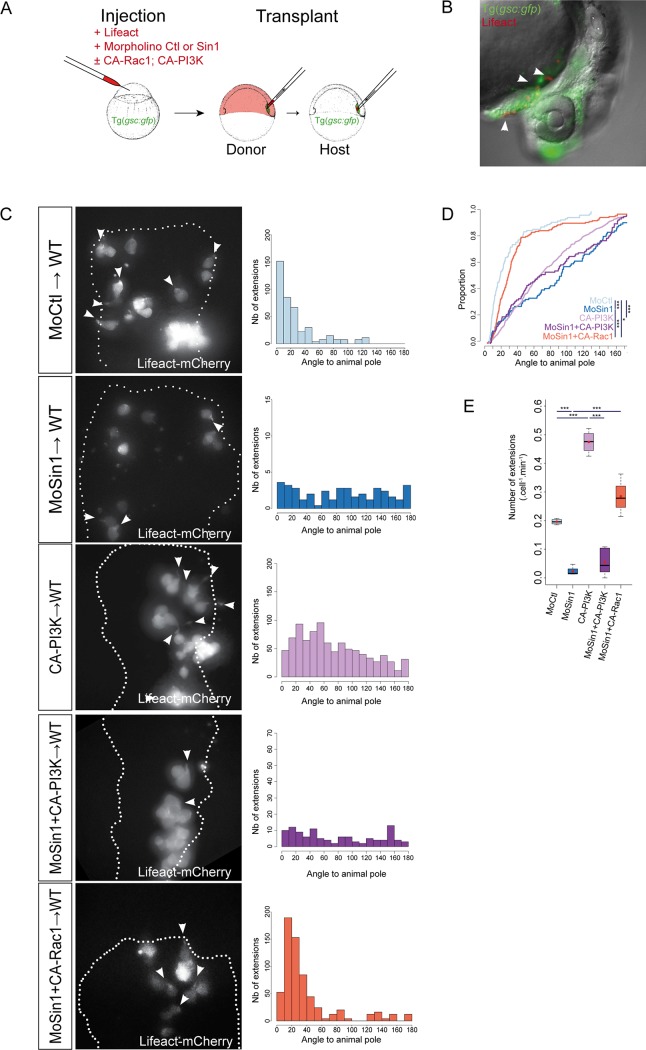
Absence of Sin1 affects protrusive activity of prechordal plate cells. (A) Diagram of the design of prechordal plate cell transplantation. Cells were labelled with Lifeact-mCherry and transplanted from shield to shield. (B) At 24 hpf, *sin1* morphant cells transplanted into wild-type prechordal plates take part to the hatching gland. (C) Cells injected with Lifeact-mCherrry RNAs and either a control morpholino, or the *sin1* morpholino, or CA-PI3K RNAs, or the *sin1* morpholino and CA-PI3K RNAs, or the *sin1* morpholino and CA-Rac1 RNAs were transplanted from shield to shield in *Tg(-1*.*8gsc*:*GFP)* embryos. Transplanted cells are within the prechordal plate (assessed by GFP expression), and contour of the plate is delineated (white dotted line). Orientations of their cytoplasmic extensions were measured relative to the animal pole and plotted as histograms (C) and cumulative plot in (D). (E) Frequencies of cytoplasmic extensions.

The TORC2 complex has been shown to control in vitro migration of several cell types. In these systems, TORC2 regulates actin dynamics. To analyse the subcellular origin of the observed prechordal plate migration defects, prechordal plate cells were grafted from control or *sin1* morphant embryos into the plate of uninjected *Tg(-1*.*8gsc*:*GFP)* embryos (Fig. 4A). The grafted cells were labelled with a Lifeact-mCherry fusion in order to visualise actin dynamics [37]. Cells injected with a control morpholino emit actin-rich extensions pointing mainly towards the animal pole, as previously described [35,38] (Fig. 4C-E; MoCtl: 0.196 extensions.min^-1^.cell^-1^; n = 112 extensions, 14 cells, 4 embryos; S5 Movie). In *sin1* morphant cells, the frequency of extension emission is drastically decreased (Fig. 4E; MoSin1: 0.02 extensions.min^-1^.cell^-1^, p = 1.3.10^–5^) and extensions are emitted in all directions (Fig. 4C-D and S5 Movie; n = 71 extensions, 46 cells, 4 embryos, p = 2.8.10^–11^). This demonstrates that Sin1 is an essential factor for formation and orientation of actin-rich cell protrusions. These transplant experiments furthermore demonstrate that Sin1 acts in a cell-autonomous manner in prechordal plate cells.

### Sin1 is required for PI3K induced protrusive activity

The PI3K pathway has been involved, possibly downstream of PDGF, in prechordal plate migration [35,38]. Prechordal plate cells expressing a dominant negative form of PI3K show a reduction in actin-rich protrusion frequency and a randomization of the remaining protrusions [38]. This phenotype resembles the one observed in *sin1* loss of function, suggesting that PI3K and Sin1 may function in the same pathway. It was furthermore demonstrated that, in HEK293T cells, the TORC2 kinase activity can be stimulated by PI3K [11], and that TORC2 is activated downstream of PI3K, for Prostaglandin E2 induced chemotaxis of mast cells [21]. These observations strongly suggested that Sin1 may function downstream of PI3K in prechordal plate migration. To test this possibility, we assessed whether Sin1 is required for PI3K induced protrusive activity. Wild-type cells expressing a constitutive form of PI3K (CA-PI3K, also known as p110CAAX) [39] were transplanted into the prechordal plate of *Tg(-1*.*8gsc*:*GFP)* embryos. As previously described [38], expression of the CA-PI3K enhances protrusion formation (Fig. 4C-E and S5 Movie, CA-PI3K: 0.47 extensions.min^-1^.cell^-1^; n = 412 extensions, 15 cells, 4 embryos, p_MoCtl/CA-PI3K_ = 5.10^–4^) and leads to their randomization (p_MoCtl/CA-PI3K_<2.2.10^–16^). However, co-injection of the *sin1* morpholino with the CA-PI3K construct, blocks the effect of activated PI3K (Fig. 4C-E and S5 Movie, MoSin1+CA-PI3K: 0.06 extensions.min^-1^.cell^-1^; n = 109 extensions, 24 cells, 5 embryos, p_CA-PI3K/MoSin1+CA-PI3K_ = 2.2.10^–6^), leading to the same phenotype as injection of the *sin1* morpholino alone (for protrusion frequency p_MoSin1/MoSin1+CA-PI3K_ = 0.22, and for protrusion orientation p_MoSin1/MoSin1+CA-PI3K_ = 0.25). This shows that Sin1 is required to mediate PI3K protrusive activity in prechordal plate cells.

### *sin1* loss of function is rescued by activated Rac1

In mammalian cell cultures, it has been shown that TORC2 can control actin dynamics through activation of Rac1 [23,40]. As for PI3K, the prechordal plate migration defects observed in absence of *sin1* strongly resemble the phenotype upon *rac1* loss of function (reduction of the number of cell protrusions and randomisation of the remaining ones; [35]), suggesting that *sin1* may regulate actin dynamics in prechordal plate through *rac1*. To address this possibility, we asked if activation of Rac1 could rescue *sin1* loss of function phenotypes. Co-injection with the *sin1* morpholino of mRNAs encoding a constitutively active form of Rac1 (CA-Rac1) restores the protrusive activity of the cells (Fig. 4E and S5 Movie; 0.285 extensions.min^-1^.cell^-1^, p_MoSin1/MoSin1+CA-Rac1_ = 0.022, p_MoCtl/MoSin1+CA-Rac1_ = 0.17). Strikingly, the orientation of protrusions is also largely rescued (Fig. 4C-D, n = 175 extensions, 8 cells, 4 embryos, p_MoSin1/MoSin1+CA-Rac1_ = 3.10^–13^, p_MoCtl/MoSin1+CA-Rac1_ = 0.014).

Altogether, these results demonstrate that *sin1* controls cell protrusive activity, and is very likely acting downstream of PI3K and upstream of Rac1.

## Discussion

In the past decade, it became clear that in addition to the well-studied TORC1 complex, Tor can form a second complex, TORC2, which differs in protein composition and in functions. TORC2 was identified as a regulator of the actin cytoskeleton in yeast, *Dictyostelium* and mammalian cell lines. However, because of the early embryonic lethality of *sin1* and *rictor* knock downs in mice, the in vivo importance of TORC2 in actin regulation and cell migration had not been addressed. Here we analysed the effect of *sin1* loss of function on early zebrafish development and provide evidence that TORC2 controls cell migration in vivo. We found that, in the absence of Sin1, speed of prechordal plate migration is reduced by half. This is due to reductions in both cell speed and cell directionality, which can be attributed to a drastic reduction in the protrusive activity of the cells, and a randomisation of their remaining cytoplasmic extensions.

Notably, despite this drastic cellular phenotype, cells lacking Sin1 migrate at a normal pace when transplanted into a wild-type host, and can later differentiate into hatching gland, the prechordal plate derivative (Fig. 4B). Their normal pace indicates the existence of non-cell autonomous effects within the plate, whereby defective cells are carried by their wild-type neighbours [27]. This is in accordance with previous analyses showing that dividing cells within the migrating prechordal plate hardly slow down, despite lacking protrusive activity during mitosis [35]. More surprisingly, in morphant embryos where all cells are affected, prechordal plate migration is slowed down, but not abolished. A similar observation has been reported for other knock-downs affecting protrusion formation [28,35,38]. Indeed, there are no known mutations that specifically and completely stop prechordal plate migration, which could be explained by two non-exclusive phenomena. First, it has been reported that, in addition to protrusions, prechordal plate cells also produce blebs, which could be responsible for part of their movement [41]. Second, it is possible that prechordal plate migration has a non-autonomous component contributed by the notochord. Notochord cells, posterior to the plate, undergo convergence and extension movements during gastrulation, and extension of the notochord may displace the plate towards the animal pole [42].

### TORC2 or Sin1 specific functions?

We used the loss of function of *sin1* to address the in vivo roles of TORC2. Are the observed phenotypes due to a loss of TORC2 function or to TORC2 independent roles of Sin1? Sin1 is a core component of the TORC2 complex, from yeast to humans, including fly and *C*. *elegans*. Even though not yet demonstrated in zebrafish, it is very likely part of the fish TORC2 complex, and it has been shown that Sin1 is crucial to TORC2 function and stability [8,9]. However, it has been shown that Sin1 can also interact with proteins unrelated to TORC2 [43–45]. We thus cannot formally exclude that the observed phenotypes may be due to TORC2-independent functions of Sin1. However, these TORC2 independent roles of Sin1 are related to stress response, not to actin regulation. A definitive role of the TORC2 complex on actin dynamics has been well documented in vitro. Our observed actin and migration defects in the prechordal plate are thus very likely due to TORC2 loss of function. Confirming this would require knocking down *rictor*, the other specific core component of the complex. Unfortunately, there seems to be three *rictor* orthologues in the zebrafish genome, complicating such loss of function approaches. Furthermore, it has been suggested that *rictor* loss of function can affect cell migration in a TORC2 independent manner [46].

### What is acting upstream of TORC2?

In vertebrates, the signaling pathways that lead to TORC2 activation are not well characterized. The best known extracellular signals that activate TORC2 are trophic factors and hormones that lead to increased phosphoinositide 3-kinase (PI3K) signaling [47,48] suggesting that PI3K may act upstream of TORC2. This has been shown to be the case for mast cell chemotaxis, where TORC2 is activated by PI3K [21]. Here, we have established that Sin1 is required to mediate PI3K effect on protrusive activity, implying that the function of TORC2 downstream of PI3K may be conserved in many migrating cells. Whether PI3K actually modulates TORC2 activity in prechordal plate cells, and if so, how it does so remains to be established. Several components of the TORC2 complex contain PH domains indicating possible regulation by PIP3 [11]. Alternatively, TORC2 activity may be regulated indirectly, or may even be independent of PI3K, as it is in Dictyostelium [19,49]. In this case, TORC2 would be necessary for PI3K induced protrusive activity, but directly activated by PI3K.

### What is acting downstream of TORC2?

In vertebrates, two main pathways have been identified linking TORC2 to actin regulation and cell migration. One is modulation of RhoA through PKC [20] and the other is modulation of Rac1, most likely through P-Rex1 (PIP3 dependent Rac exchange factor) [23]. The similarity between the *sin1* and *rac1* loss of function phenotypes, and, more importantly, the fact that activated Rac1 can rescue *sin1* loss of function, strongly suggests that, in prechordal plate cells, the effect of TORC2 on actin dynamics is mediated by Rac1. Whether this involves P-Rex1 remains to be established.

It is also possible that TORC2 modulates RhoA through PKC, and hence controls cell polarity and protrusive activity [20,50]. Unfortunately, we could not find phospho-specific antibodies for the different PKCs in zebrafish, which prevented the direct testing of this hypothesis. However we have shown that RhoA loss of function has very limited effect on prechordal plate cell migration and dynamic [35], pleading against the involvement of this PKC-RhoA pathway in these cells.

### Orientation of actin-rich protrusions

*sin1* loss of function leads to a drastic reduction in the number of cell protrusions as well as to the randomisation of the remaining protrusions, suggesting that TORC2 is required for orientation of protrusions. Similar observations in absence of PI3K activity, as well as the observation of anterior accumulations of PIP3, have led to propose that PI3K controls protrusion orientation [35]. However, we surprisingly observed that overexpression of an activated form of Rac1 not only rescues protrusion formation but also rescues their orientation. This leads to two conclusions. i) There are probably two different mechanisms controlling protrusion formation, a minimal, Rac1 independent, mechanism which would account for the few randomised protrusions observed in absence of TORC2, and a Rac1 dependent mechanism, responsible for oriented protrusions. ii) Orientation of this Rac dependent system does not rely on localised activation of Rac1, since ubiquitous expression of activated Rac1 rescues orientation. Orientation could be achieved through localised recruitment of Rac1 or of activated Rac1. Analysing localisation of Rac1 and activated Rac1 would address these possibilities. Whether this recruitment, and thus protrusion orientation, is dependent on PI3K is also a question of interest and might be determined by overexpression of activated Rac1 in absence of PI3K.

## Conclusion

In the past few years, TORC2 was shown to control actin dynamics and cell migration in a rapidly growing number of in vitro systems. We demonstrated here that it plays such a role in vivo and is key to proper embryo development. Further work, both in vitro and in vivo, will be necessary to identify its upstream activators and to clarify its link to actin regulators such as Rac1. Using conditional knockouts and/or mosaic embryos will also be of major interest to analyse the roles of TORC2 in controlling migrations taking place later in development.

## Materials and Methods

### Ethics statement

All animal studies were done in accordance with the guidelines issued by the French Ministry of Agriculture and have been submitted to Paris ethical committee no. 3 (headed by J. Yelnik, INSERM U679, Paris). The committee validated the studies but estimated that no formal approval was required, since experiments on embryos before the last third of their development are not regarded as experiments on animals, according to the article R. 214–88 of the decree 2013–118 (“N’entre pas dans le champ d’application les formes embryonnaires des vertébrés ovipares avant le dernier tiers de leur développement normal”).

The facility in which adults were maintained has received the approval of veterinary services (Approval N° A75-05-32). ND has received an authorisation to experiment on Vertebrates (N° A-75-1832). All efforts were made to ensure welfare of the animals.

### Animals

Embryos were obtained by natural spawning of WT or *Tg(-1*.*8gsc*:*GFP)ml1* adult fish [34], and staged according to [26].

### Sin1 isolation and cloning

Sin1 ORF was amplified by RT-PCR on mRNA extracts from gastrulae and 24hpf embryos, using primers designed according to the available genomic sequence (Zv9) (Ensembl: ENSDARG00000091777).

Primers used were: 5’-ATCTCTAGATTACTGGCCTGACTTTTTGTCC-3’ and 5’-ATCGAATTCATGGCTTTCCTGGACAACCC-3’.

The ORF was then cloned using Gateway technology in pSPE3 [51] and in pDest2-mCherry [52].

### Morpholino and mRNA injections

mRNAs for injection were synthetized in vitro using SP6 promoter (mMESSAGE mMACHINE SP6, Ambion) from pCS2-Lifeact-mCherry (80pg), pCS2-H2B-mCherry (100pg) [35], pCS2-CA-PI3K (p110CAAX) (120pg) [38], pCS2-CA-Rac1 (2pg) [53] and pCS2-Sin1-mCherry (80pg) (cloned by In-Fusion, Clonetch). For *sin1* loss of function, two independent translation blocking morpholinos were used, with their 5-mismatches controls. One was targeted at the ATG start codon (Mo-ATG: 5’-GGCCGGGTTGTCCAGGAAAGCCAT-3’; Ctl 5-mismatches: GcCCcGGTTcTCCAGcAAAGCgAT). The second was targeted at 5’-UTR sequences (Mo-5’UTR: 5’-AGCCCCTCGTCCCTCTTCTTCTGTC-3’; Ctl 5-mismatches: 5’-AGCCCgTCcTgCCTCTTCTTgTcTC-3’). At high doses (1.4mM for Mo-ATG and 1mM for Mo-5’-UTR), both morpholinos lead to an early embryonic lethality. All analyses were thus performed at lower doses, likely creating a hypomorphic situation (1mM for Mo-ATG and 0.3mM for Mo-5’-UTR). Both morpholinos led to identical phenotypes.

### Whole-mount in situ hybridisation

In situ hybridisation was performed following standard protocols [54] using *sin1* (in pSPE3), *sox17* [31], *ta* [30], *tbx6* [46] and *otx2* [29] probes. All probes were digoxygenin labelled.

### Cell transplantation

*Tg(-1*.*8gsc*:*GFP)* embryos were injected at the 1 cell stage with 80pg of Lifeact-mCherry mRNA and either with MoCtl or MoSin1. At the onset of gastrulation, small cell groups were transplanted from shield to shield. The prechordal plate identity of transplanted cells was assessed by their GFP expression. Cellular extension orientations were measured as the angle between the cell protrusion and the direction of the animal pole.

### Time-lapse imaging and track analyses

Imaging of cell transplants was either done on a Nikon spinning disk equipped with an Evolve camera (Photometrics), or an Axioplan 2 microscope (Zeiss) equipped with a CoolSNAPcf camera (Photometrics) and MetaVue software (Molecular Devices).

For detailed analysis of prechordal plate cells speeds and trajectories, dechorionated embryos were mounted in 0.2% agarose in embryo medium. Z-stacks were collected at 2 min intervals on a thermostated Nikon spinning disk. Nuclei were first automatically tracked in 3D using Imaris software (Bitplane Scientific). Tracks were then manually corrected and validated. Data were then processed in Matlab (Mathworks) using custom made routines as described in [35], to extract, in particular, instantaneous speed and persistence. Instantaneous speed is the displacement vector between two consecutive time points, divided by the time interval. Persistence was defined as the ratio between net displacement (distance between initial and final position) and total displacement (sum of displacements between each time points), computed over 10-min intervals. For 3D representations, for each embryo, tracks at different times points were registered relative to the position of the front of the plate (for x axis) and, between embryos, tracks were registered relative to the centre of the plate (y and z axes). Outliers were removed according to Chauvenet criterion. Data were then interpolated (TriScatteredInterp), and instant speed (norm and direction) was computed on a 3D mesh grid to estimate its value at all positions of the plate.

### Illustrations and statistical analyses

Images were processed with ImageJ and Adobe Photoshop. Figs. were assembled with Adobe InDesign. 3d colour coded quiver plots were produced in Matlab, using the quiver3Dpatch (available on Matlab Central). Histograms and statistical analyses were performed in R (http://www.r-project.org/). T-test was used to compare means when a single measurement was done on each embryo. When multiple measurements where done on the same embryo, linear mixed-effects models were used to take into account resampling of the same statistical unit. Kolmogorov-Smirnov test was used to compare non-circular angle distributions. Power cannot be computed for linear mixed-effects model and was thus estimated by computing power of a t-test on the same samples with half of the observed effect size (pwr.t2n.test from the ‘pwr’ package in R; Cohen’s effect size).

## Supporting Information

S1 Fig *sin1* is expressed ubiquitously during the first 24 hours of development. In situ hybridisation of *sin1* probe (A-E), or a sense probe used as a control (A’-E’), at the 1-cell stage (A), in blastula (sphere stage; B), at the onset of gastrulation (shield stage; C), at mid-gastrulation (75% epiboly; D) and at 24-hpf (E). *sin1* appears maternally inherited and ubiquitously expressed. A stronger signal is observed in the forming embryonic axis during gastrulation (black arrowheads in C and D)
(TIF)

S1 Movie Prechordal plate migrates slower in *sin1* morphant embryos. *Tg(-1*.*8gsc*:*GFP)* embryos injected with a control morpholino (left panel) or *sin1* morpholino (right panel). Position of the front of the prechordal plate is delineated (dashed line).
(MP4)

S2 Movie 3D tracking of prechordal plate cells migration in a control embryo. *Tg(-1*.*8gsc*:*GFP)* embryo injected with a control morpholino. Prechordal plate cells (green) were tracked by following Histone-2B-mCherry labelled nuclei.
(MP4)

S3 Movie 3D tracking of prechordal plate cells migration in a *sin1* morphant embryo. *Tg(-1*.*8gsc*:*GFP)* embryo injected with *sin1* morpholino. Prechordal plate cells (green) were tracked by following Histone-2B-mCherry labelled nuclei.
(MP4)

S4 Movie *sin1* loss of function does not alter endodermal cell migration. Endodermal cells were tracked in *Tg(-1*.*8gsc*:*GFP)* embryos injected with a control morpholino (left panel) or *sin1* morpholino (right panel).
(MP4)

S5 Movie Sin1 controls formation of cell protrusions, through Rac1. Prechordal plate cells injected with Lifeact-mCherrry RNAs and a control morpholino, or the *sin1* morpholino, or CA-PI3K RNAs, or the *sin1* morpholino and CA-PI3K RNAs, or the *sin1* morpholino and CA-Rac1 RNAs were transplanted into the prechordal plate of untreated *Tg(-1*.*8gsc*:*GFP)* embryos. The frequency and orientation of cytoplasmic extensions were monitored.
(MP4)
