# Supplementary figures and images for: The TORC2 Component, Sin1, Controls Migration of Anterior Mesendoderm during Zebrafish Gastrulation
Source: PLoS One. 2015 Feb 24;10(2):e0118474. doi: 10.1371/journal.pone.0118474 (PMC4339552; doi:10.1371/journal.pone.0118474)

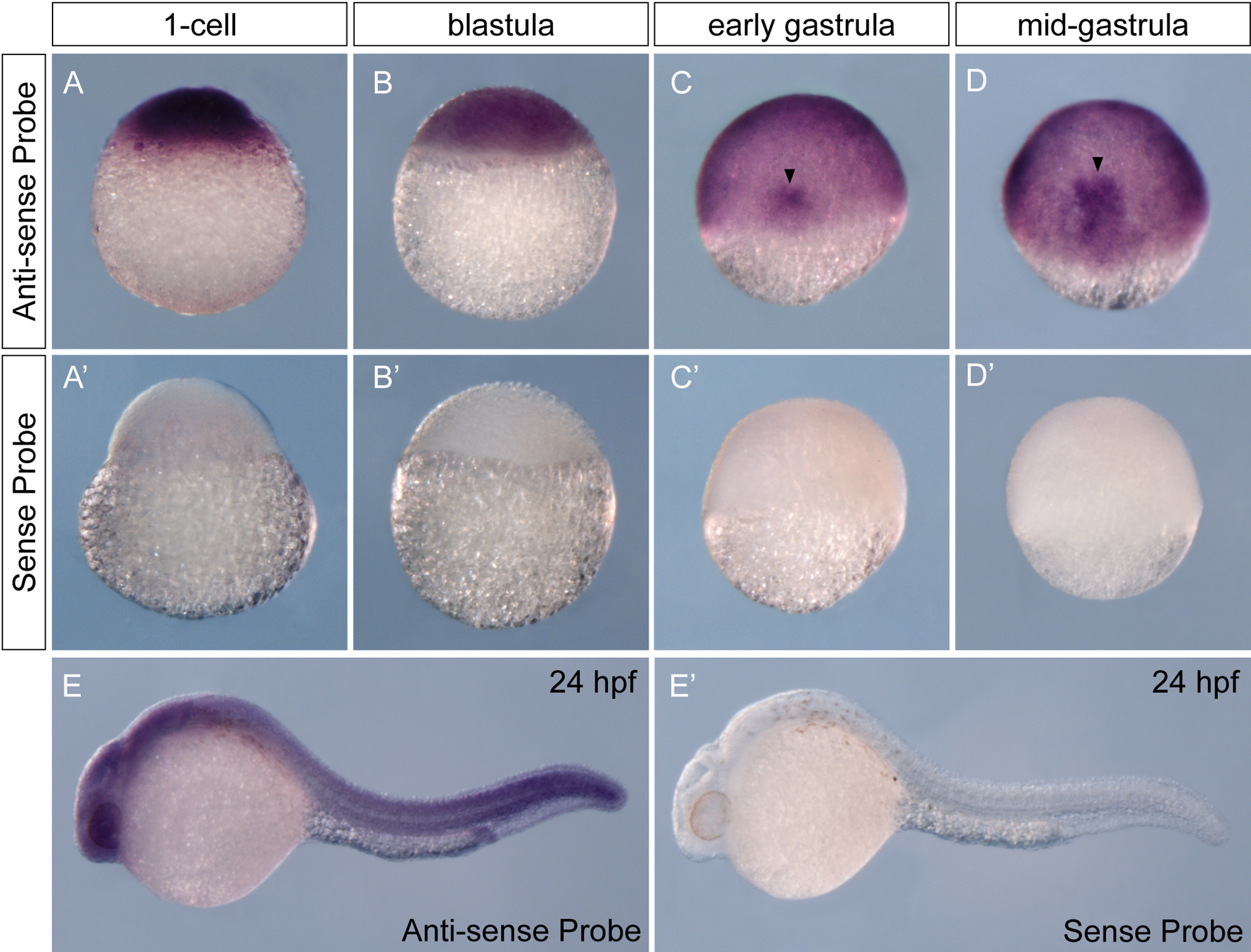

Supplement: S1 Fig — In situ hybridisation of sin1 probe (A-E), or a sense probe used as a control (A’-E’), at the 1-cell stage (A), in blastula (sphere stage; B), at the onset of gastrulation (shield stage; C), at mid-gastrulation (75% epiboly; D) and at 24-hpf (E). sin1 appears maternally inherited and ubiquitously expressed. A stronger signal is observed in the forming embryonic axis during gastrulation (black arrowheads in C and D) (TIF) [file pone.0118474.s001.tif]
